# Supplementary material for: Kidney cytosine methylation changes improve renal function decline estimation in patients with diabetic kidney disease
Source: Nat Commun. 2019 Jun 5;10:2461. doi: 10.1038/s41467-019-10378-8 (PMC6549146; doi:10.1038/s41467-019-10378-8)
Supplement: Supplementary file 1 — Supplementary Information [file 41467_2019_10378_MOESM1_ESM.pdf]

Supplementary Information for:

Kidney cytosine methylation changes improve renal function decline estimation in patients with diabetic kidney disease

Caroline Gluck[1,2], Chengxiang Qiu*[1], Sang Youb Han*[3], Matthew Palmer[4], Jihwan Park[1], Yi-An Ko[1,5], Yuting Guan[1], Xin Sheng[1], Robert L Hanson[6], Jing Huang[7], Yong Chen[7], Ae Seo Deok Park[1], Maria Concepcion Izquierdo[1], Ioannis Mantzaris[8], Amit Verma[8], James Pullman[9], Hongzhe Li[7], Katalin Susztak[1,5]

*Contributed equally

1. Department of Medicine, Renal Electrolyte and Hypertension Division, University of Pennsylvania, Philadelphia, PA, USA.
2. Department of Pediatrics, Division of Nephrology, The Children's Hospital of Philadelphia, Perelman School of Medicine, University of Pennsylvania, Philadelphia, PA, USA.
3. Division of Nephrology, Department of Internal Medicine, Inje University College of Medicine, Goyang, Korea.
4. Department of Pathology and Laboratory Medicine, Perelman School of Medicine, University of Pennsylvania, Philadelphia, USA.
5. Department of Genetics, University of Pennsylvania, Philadelphia, PA, USA.
6. Diabetes Epidemiology and Clinical Research Section, National Institute of Diabetes and Digestive and Kidney Diseases, Phoenix, AZ, USA.
7. Department of Biostatistics and Epidemiology, Center for Clinical Epidemiology and Biostatistics, School of Medicine, University of Pennsylvania Perelman, Philadelphia, PA, USA.
8. Department of Medicine, Albert Einstein College of Medicine, Bronx, NY, USA.
9. Department of Pathology Montefiore Medical Center, Bronx, NY, USA

Correspondence:

Katalin Susztak MD, PhD
Perelman School of Medicine, University of Pennsylvania
12-123 Smilow Center for Translational Research
3400 Civic Center Blvd
Philadelphia, PA 19104
215 898 2009
ksusztak@pennmedicine.upenn.edu

Supplementary Information

Supplementary Figure 1. Principal Component Analysis for the primary cohort (n=91)

Supplementary Figure 2. Association between cytosine methylation changes and baseline estimated GFR

Supplementary Figure 3. Principal Component Analysis for the replication kidney cohort (n=85)

Supplementary Figure 4. Ingenuity Pathway Analysis (IPA) for top probes associated with interstitial fibrosis

Supplementary Figure 5. Functional enrichment of 65 replicated probes associated with interstitial fibrosis in tissue specific enhancer regions

Supplementary Figure 6. For top probe, cg20597486, we completed experiments to validate the methylation changes as measured by the Illumina Infinium 450K arrays

Supplementary Figure 7. Correlation of top replicated probe methylation levels with interstitial fibrosis and Cis-gene expression levels

Supplementary Figure 8. Distribution of (a) unadjusted eGFR slope and (b) adjusted eGFR

Supplementary Figure 9. Association between eGFR slope and baseline eGFR and interstitial fibrosis

Supplementary Figure 10. Ingenuity Pathway Analysis (IPA) for top probes associated with CKD progression

Supplementary Figure 11. Correlation of top probes that improve CKD progression model methylation levels with Cis-gene expression levels

Supplementary Figure 12. Principal Component Analysis for the replication blood cohort (n=115)

Supplementary Table 1. Clinical variables associated with degree of interstitial fibrosis

Supplementary Table 2. Demographic and clinical characteristics of replication data cohorts

Supplementary Table 3. Histological characteristics of replication cohort

Supplementary Table 4. Gene Ontology for top methylation probes associated with degree of interstitial fibrosis

Supplementary Table 5. Demographic and clinical characteristics of sub-cohorts

Supplementary Table 6. Longitudinal follow up data by chronic kidney disease (CKD) stage

Supplementary Table 7. Variables associated with eGFR slope

Supplementary Table 8. Progression model with replicated fibrosis-associated probes

Supplementary Table 9. Gene Ontology for top methylation probes that improve model of kidney function decline

Supplementary Figure 1.

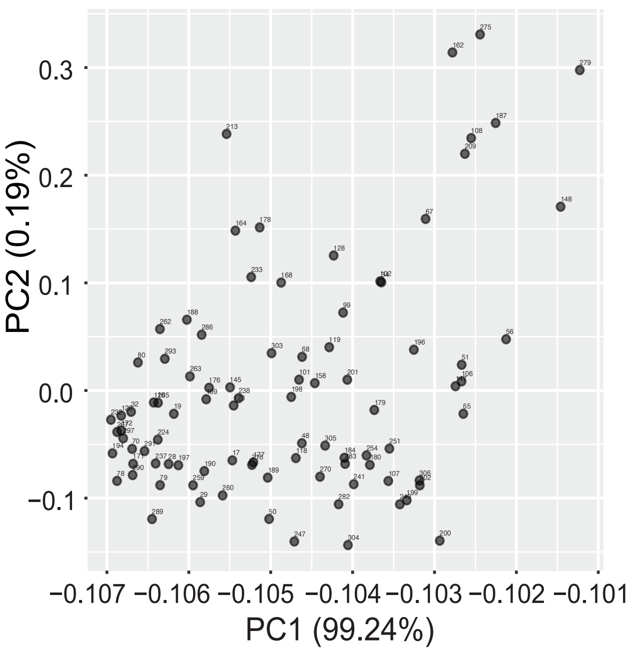

Supplementary Figure 1. Principal Component Analysis for the primary cohort (n=91).

Supplementary Figure 2.

a.

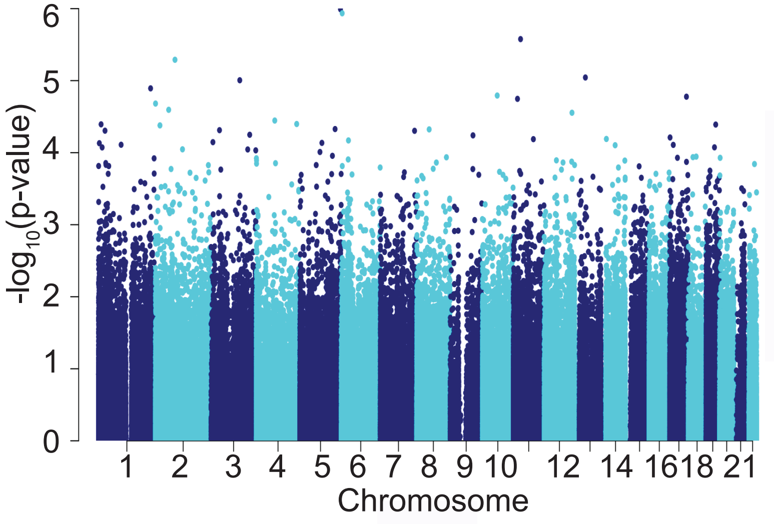

b.

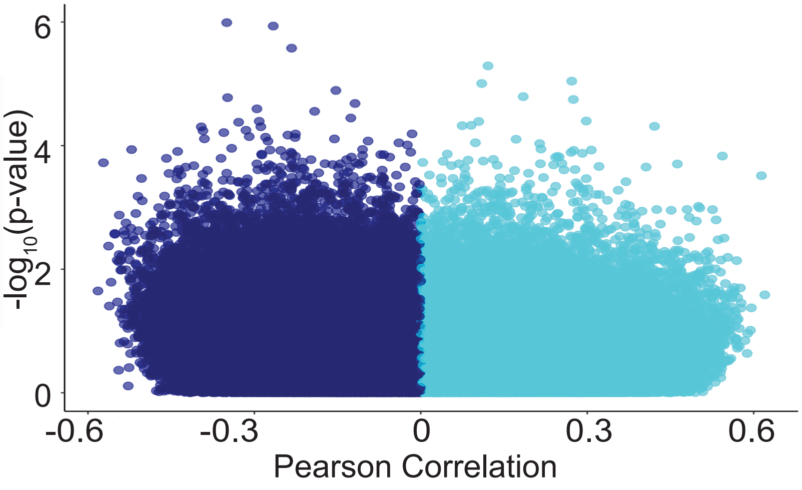

Supplementary Figure 2. Association between cytosine methylation changes and baseline estimated GFR
a.Manhattan plot of eGFR associated methylation changes. The x-axis represents the genomic location of the probe, while the y-axis is the negative base 10 log of the p- value. (The association between the methylation level of 321,473 probes and eGFR was studied using a linear regression models adjusted for age, gender, race, diabetes, hypertension, batch, bisulfite conversion, and lymphocytic infiltrate).
b.Volcano plot depicting the association between eGFR and methylation changes. . The x-axis represents the Pearson correlation coefficient of each probe with eGFR. The y-axis is the negative base 10 log of the p-value each probe associated with eGFR.

Supplementary Figure 3.

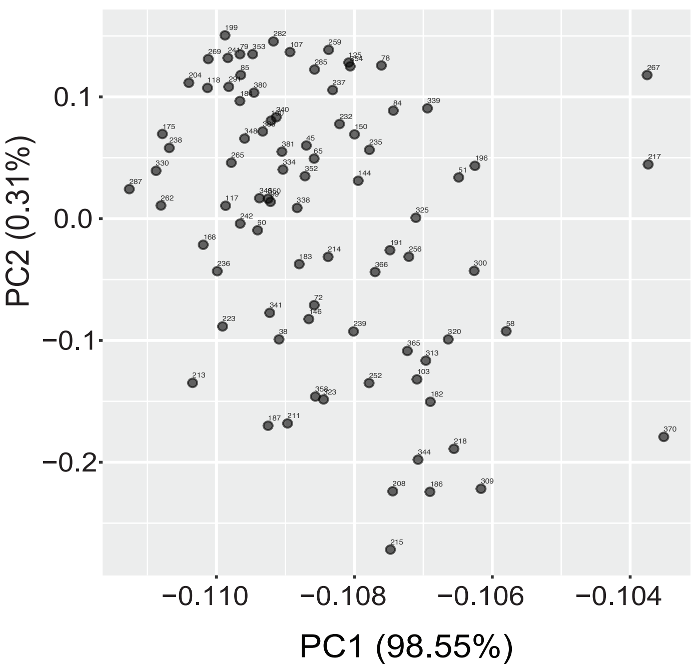

Supplementary Figure 3. Principal Component Analysis for the replication kidney cohort (n=85).

Supplementary Figure 4.

a.

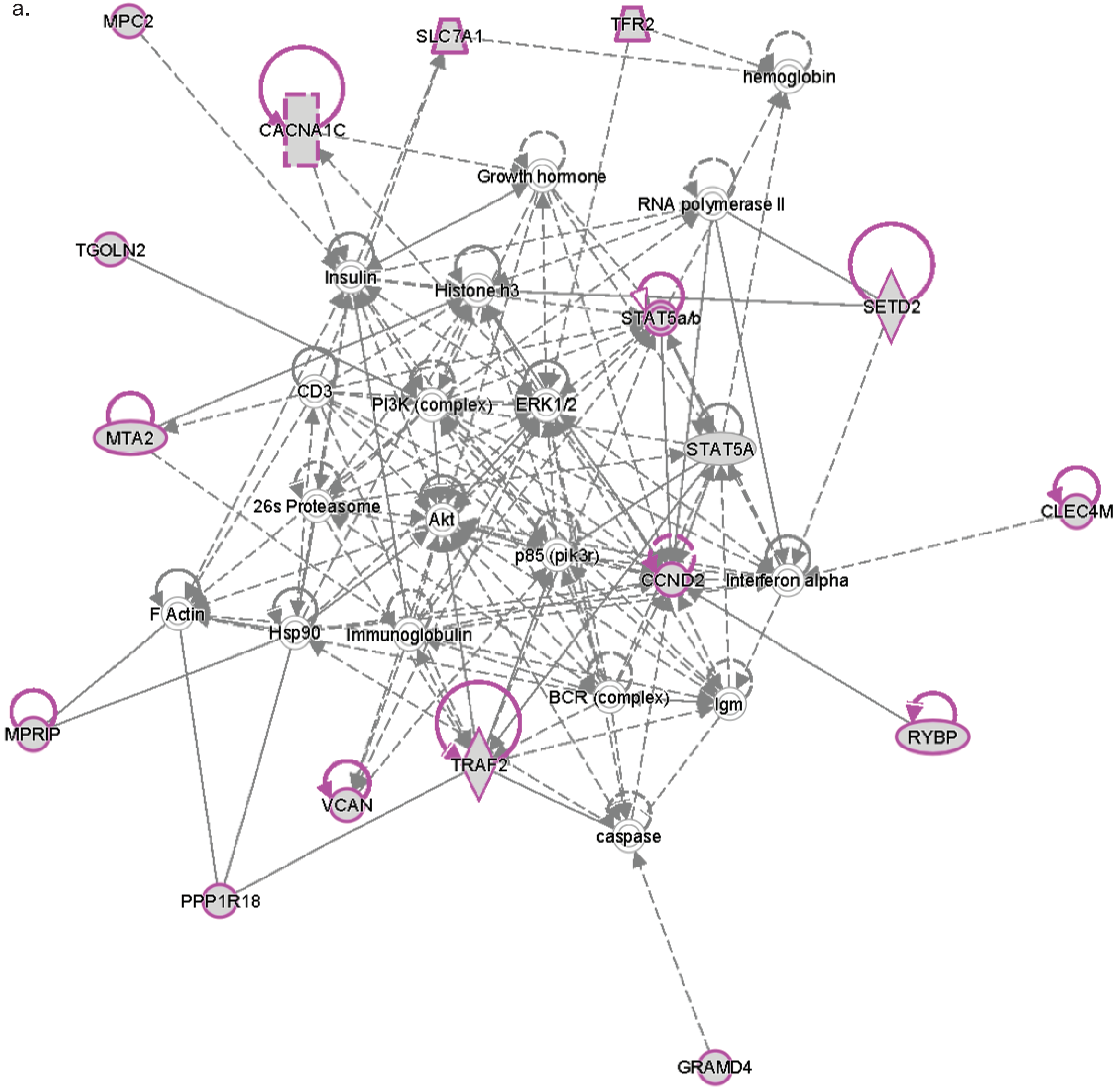

Supplementary Figure 4 continued.

b.

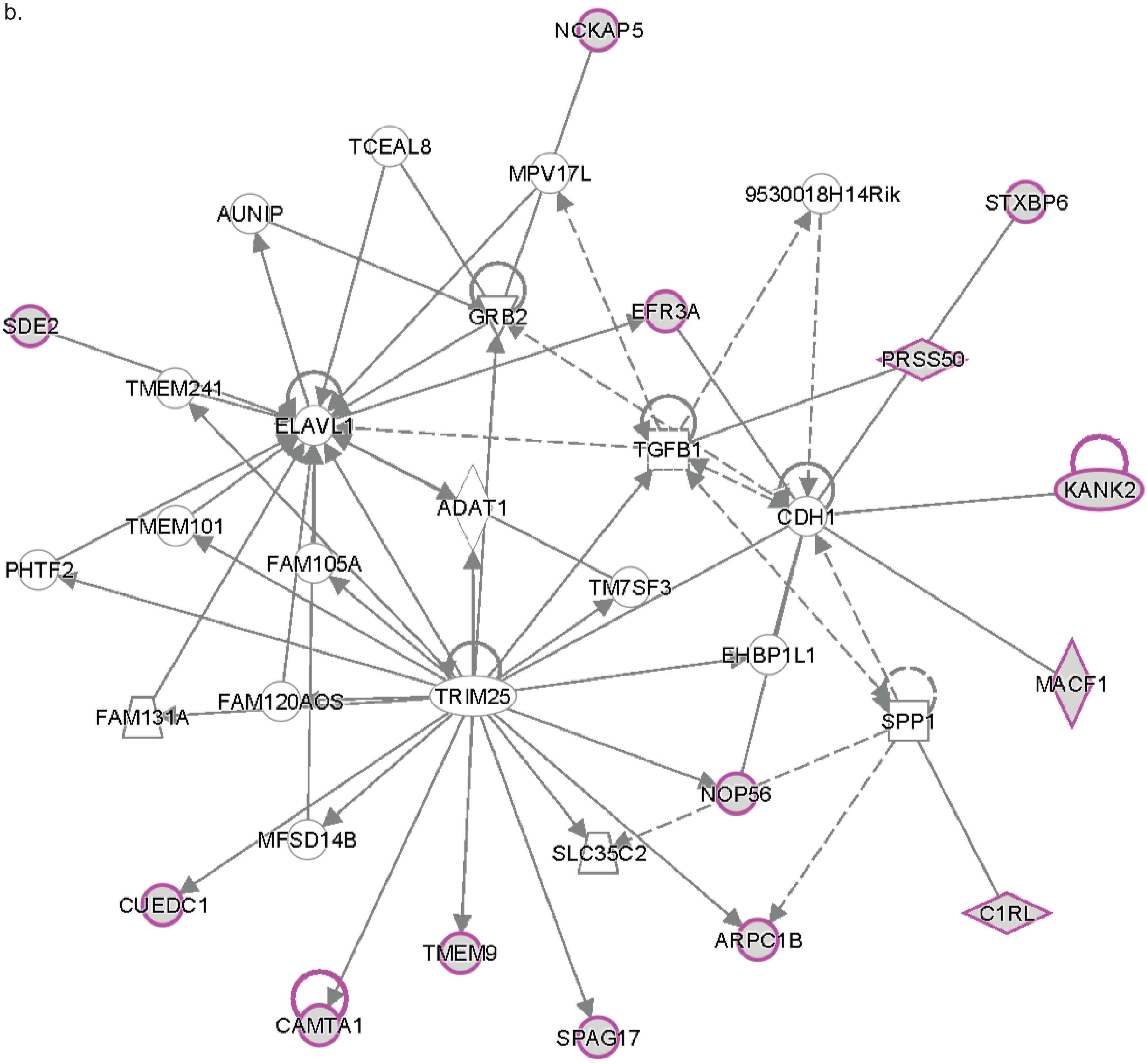

Supplementary Figure 4. Ingenuity Pathway Analysis (IPA) for top probes associated with interstitial Fibrosis. Using proximity matching we identified list of genes differentially methylated which are highlighted

a) Newtork associated with Cell Death and Survival, Increased Levels of Red Blood Cells, Connective Tissue Development and Function. Score 36.

b) Network associated with Cellular Development, Cellular Movement, Reproductive System Development and Function. Score 31.

Supplementary Figure 5.

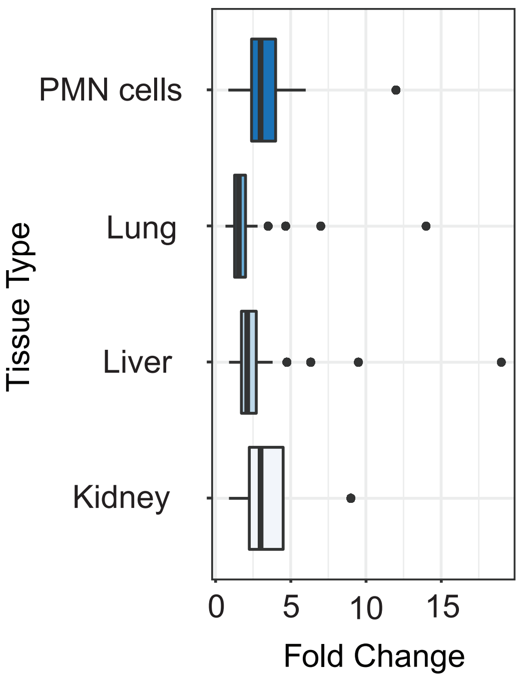

Supplementary Figure 6.

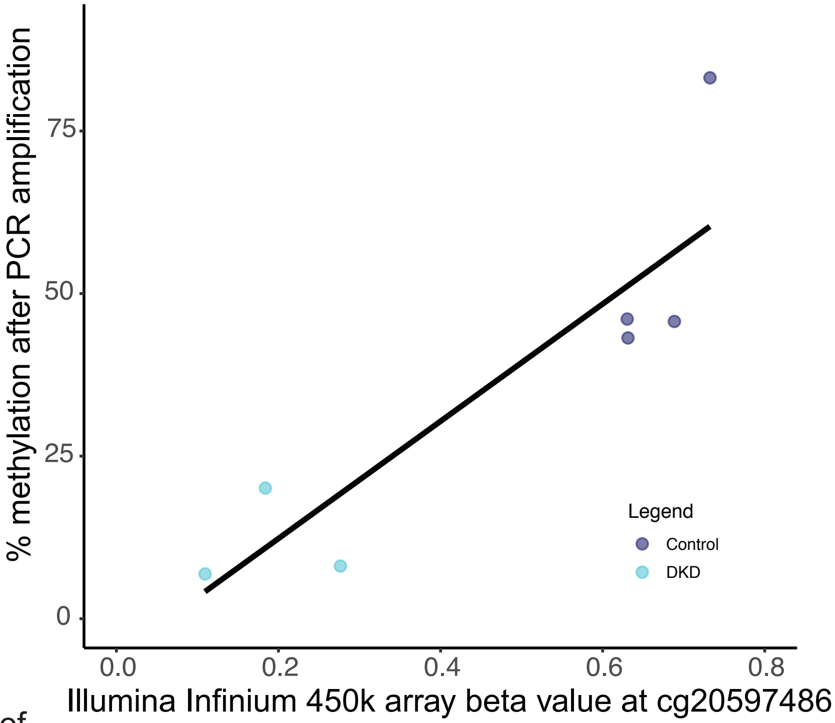

Supplementary Figure 5. Functional enrichment of 65 replicated probes associated with interstitial fibrosis in tissue specific enhancer regions. Fold change compared observed number of significant probes located in each tissue specific enhancer region to the distribution of random 65 probes selected 10,000 times from the background of all array probes used in the regression analysis (n=321,473). Median fold change for kidney enhancer was 4.5. Median fold change for liver enhancer was 2.1. Median fold change for lung was 1.6. Median fold change for polymorphonuclear (PMN) cells was 3.0. (Center line, median fold change; box limits, upper and lower quartiles; whiskers, 1.5x interquartile range).

Supplementary Figure 6. For top probe, cg20597486, we completed experiments to validate the methylation changes as measured by the Illumina Infinium 450K arrays. For 3 samples with diabetic kidney disease (DKD) and 4 control samples, we microdissected the human kidney samples and isolated the tubule compartment DNA. DNA was bisulfite converted, amplified with PCR, and transformed into bacteria. 15 colonies were selected per sample and the PCR segment was sequenced. Bisulfite converted sequences were compared with genomic DNA sequence using QUMA: quantification tool for methylation analysis. Pearson correlation coefficient between Illumina Infinium 450k array beta value and percent measure methylation for this loci was 0.88 (p-value = 0.0083).

Supplementary Figure 7.

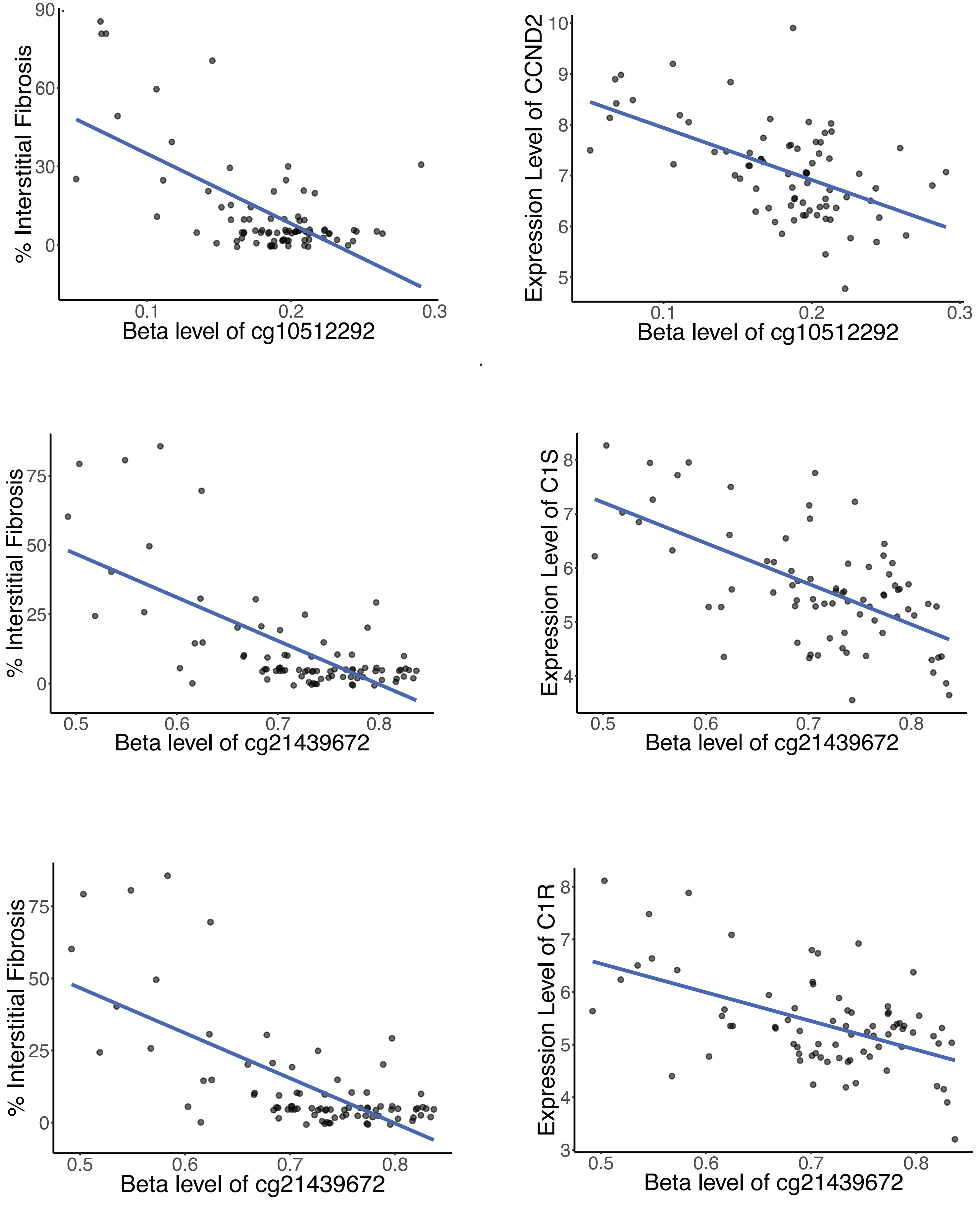

Supplementary Figure 7 continued.

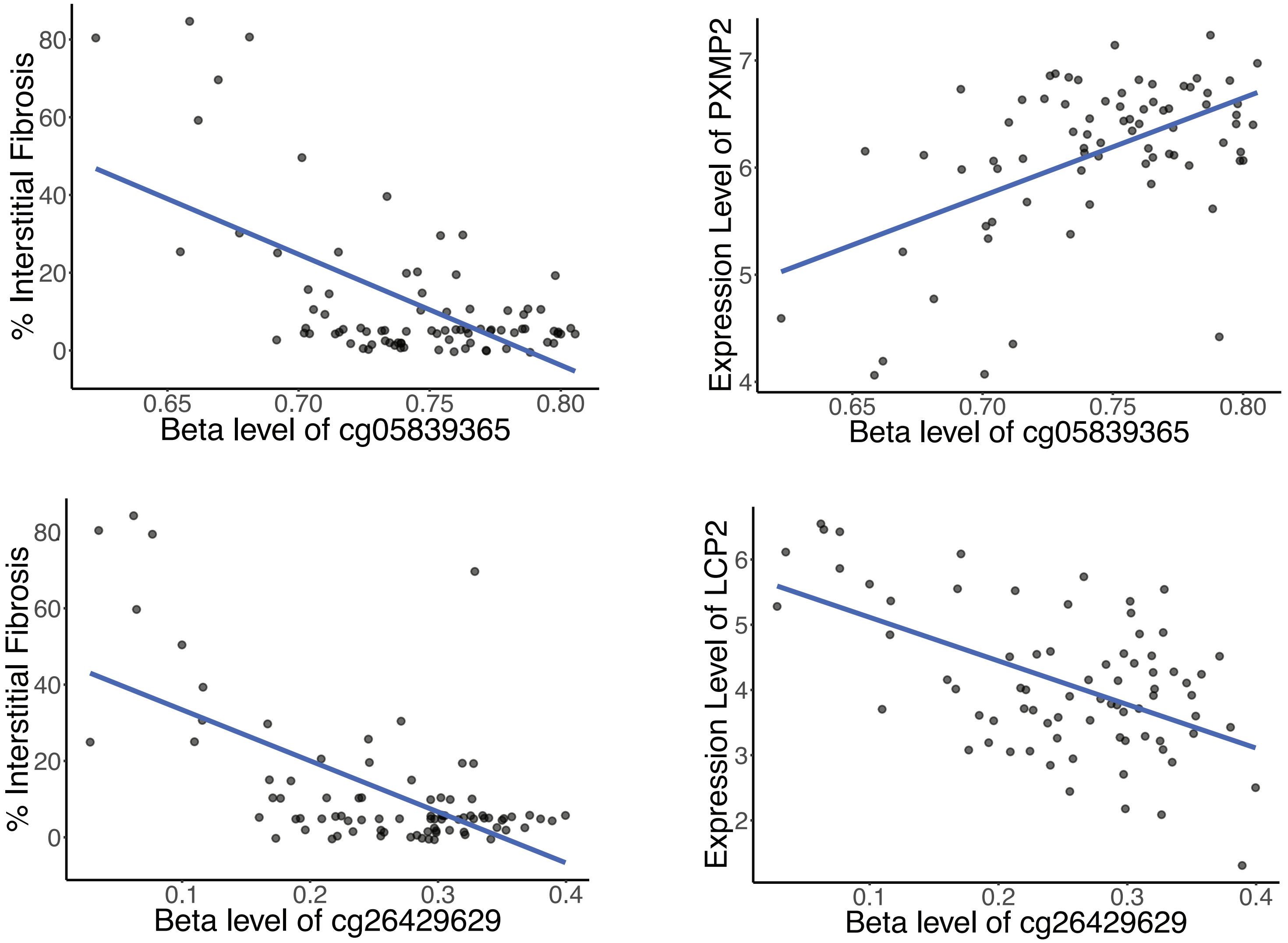

Supplementary Figure 7. Correlation of top replicated probe methylation levels with interstitial fibrosis and Cis-gene expression levels.

Supplementary Figure 8.

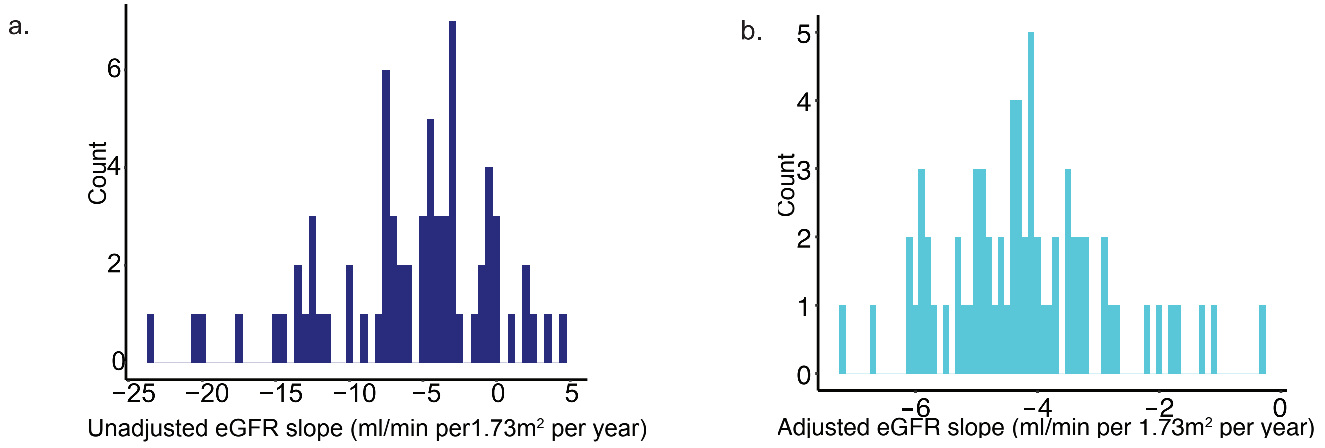

Supplementary Figure 8. Distribution of (a) unadjusted eGFR slope and (b) adjusted eGFR

Supplementary Figure 9.

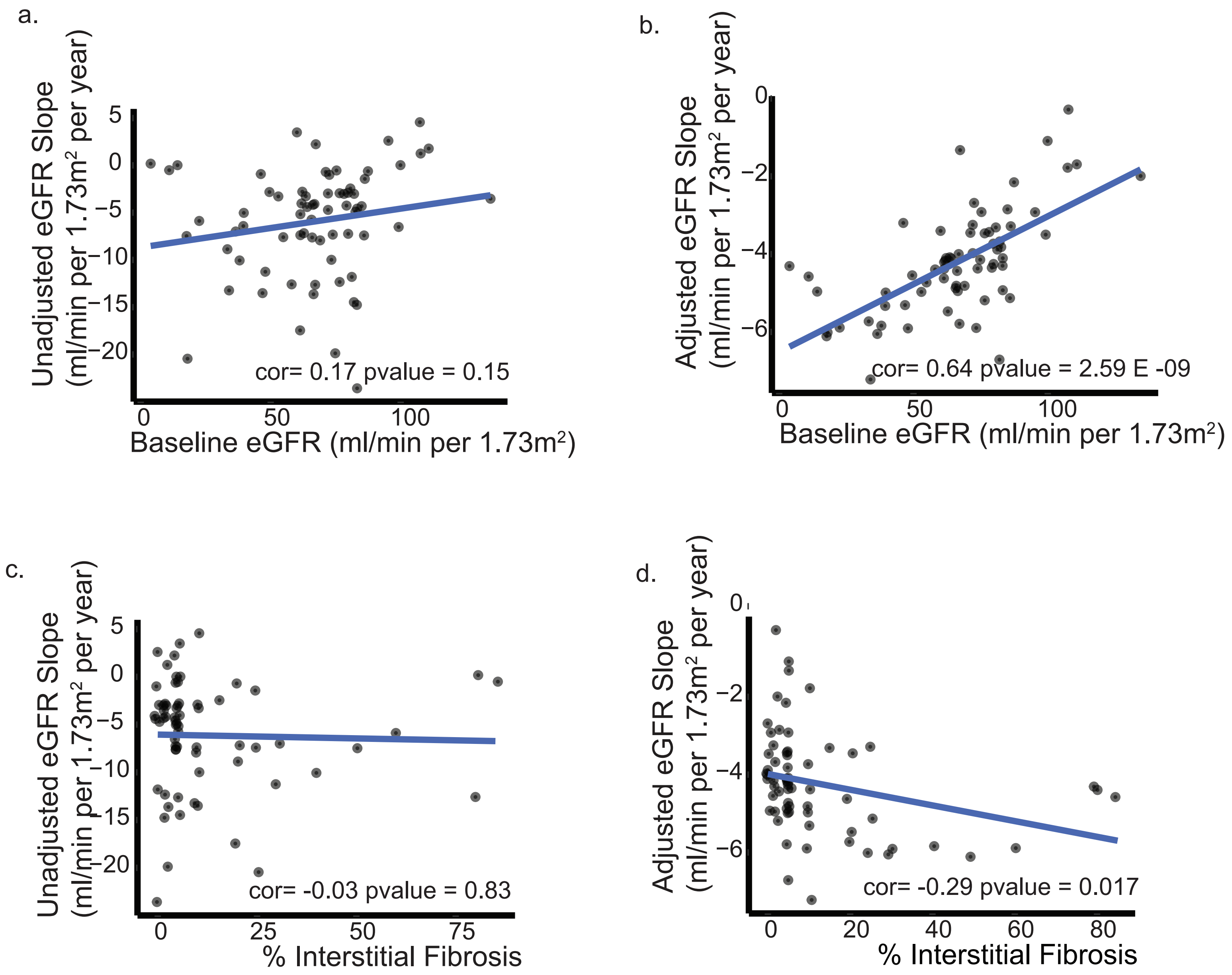

Supplementary Figure 9. Association between eGFR slope and baseline eGFR and interstitial fibrosis.
a.Unadjusted eGFR slope is not significantly associated with baseline eGFR in the primary data set using pearson correlation test. Unadjusted correlation = 0.17 (pvalue = 0.15).
b.Adjusted eGFR slope is significantly associated with baseline eGFR in the primary data set using pearson correlation test. Unadjusted correlation = 0.64 (pvalue = 2.59 e-09).
c.Unadjusted eGFR slope is not significantly associated with percent interstitial fibrosis in the primary data set using pearson correlation test. Unadjusted correlation = -0.03 (pvalue = 0.83).
d.Adjusted eGFR slope is significantly associated with percent interstitial fibrosis in the primary data set using pearson correlation test. Unadjusted correlation = -0.29 (pvalue = 0.017).

Supplementary Figure 10.

a.

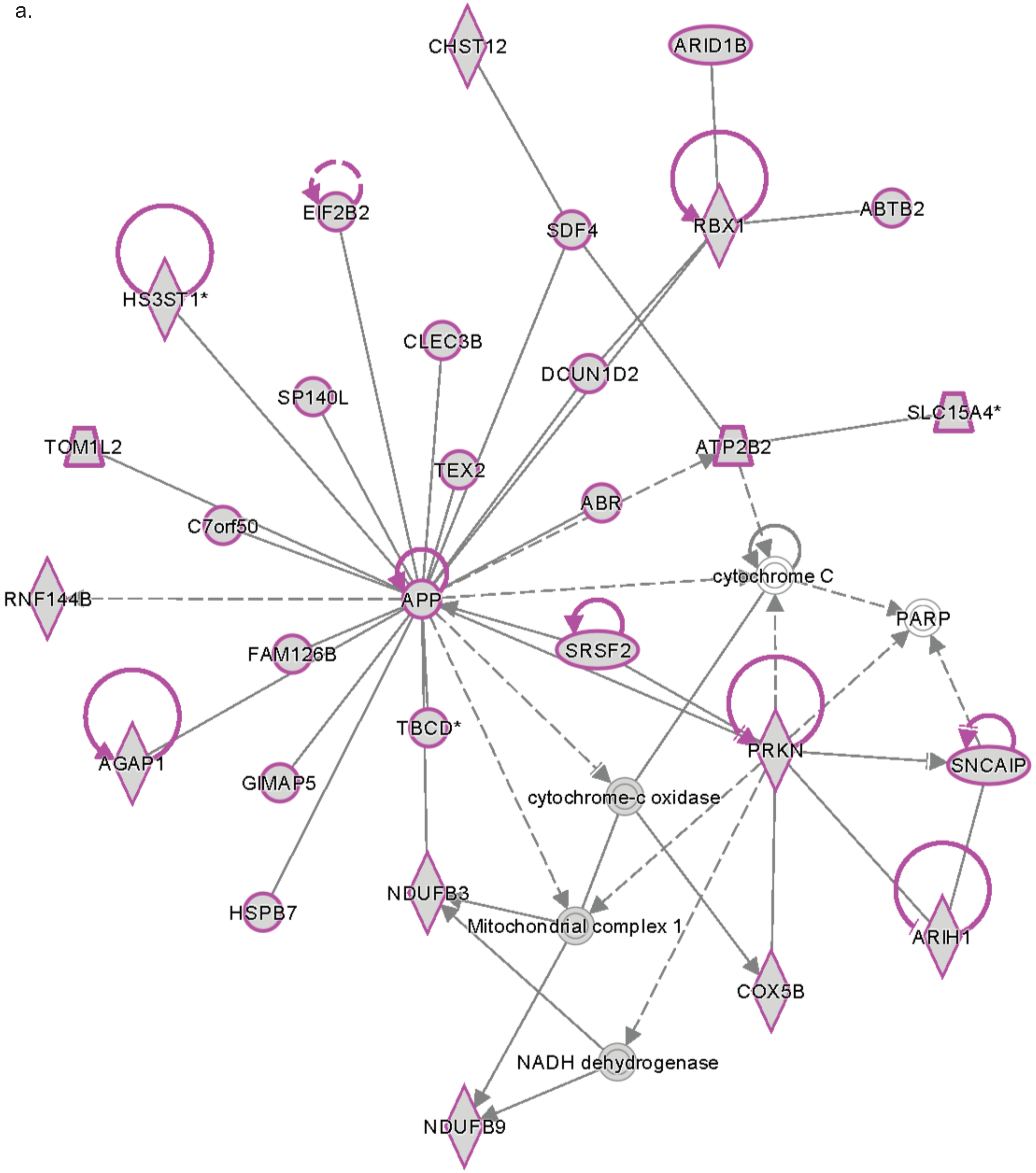

Supplementary Figure 10 continued.

b.

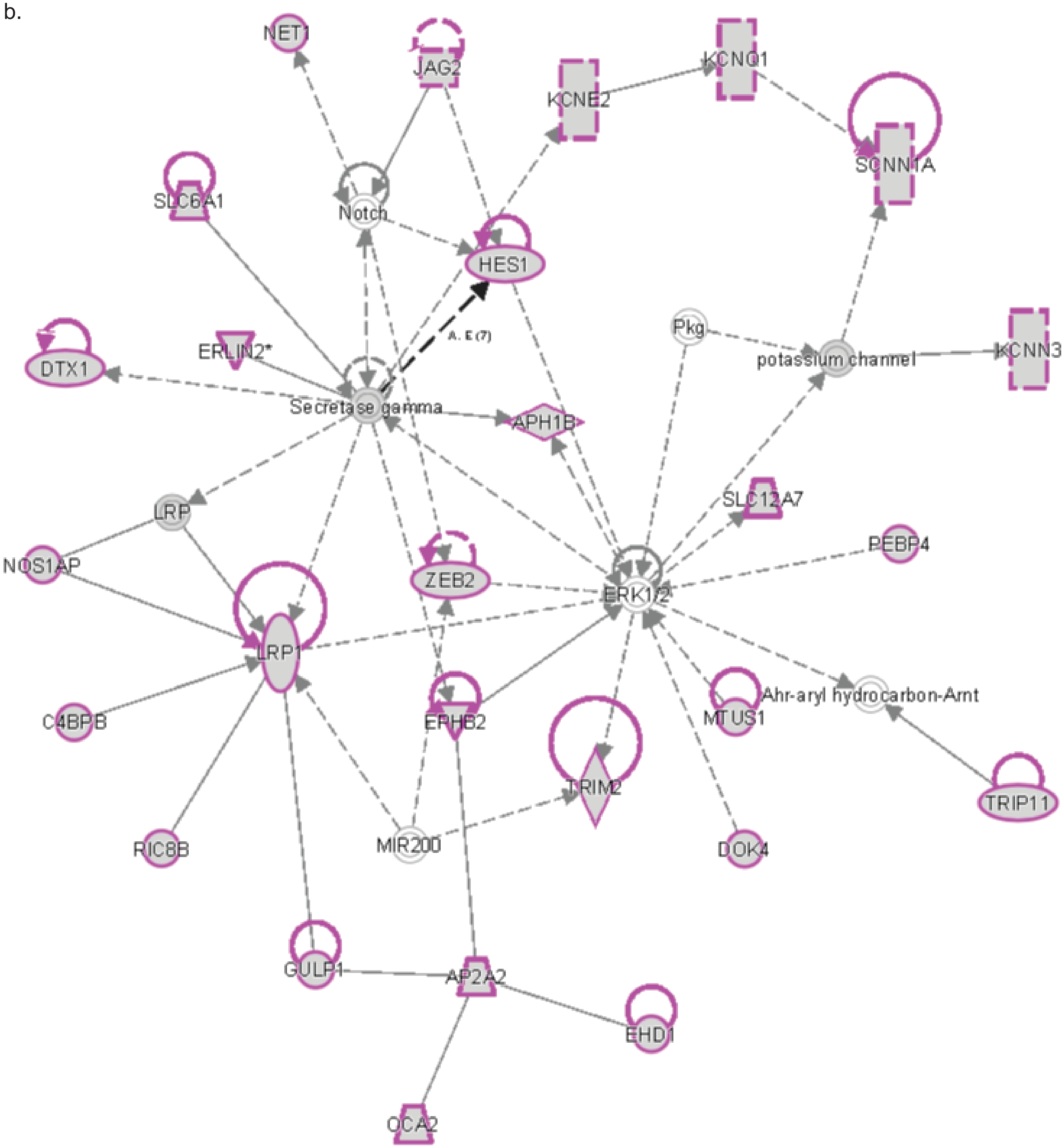

Supplementary Figure 10. Ingenuity Pathway Analysis (IPA) for top probes associated with CKD progression. Using proximity matching we identified list of genes differentially methylated which are highlighted

a) Newtork associated with Cell-To-Cell Signaling and Interaction, Small Molecule Biochemistry, Neurological Disease. Score 54.

b) Network associated with Auditory Disease, Auditory and Vestibular System Development and Function, Cell Morphology. Score 46.

Supplementary Figure 11.

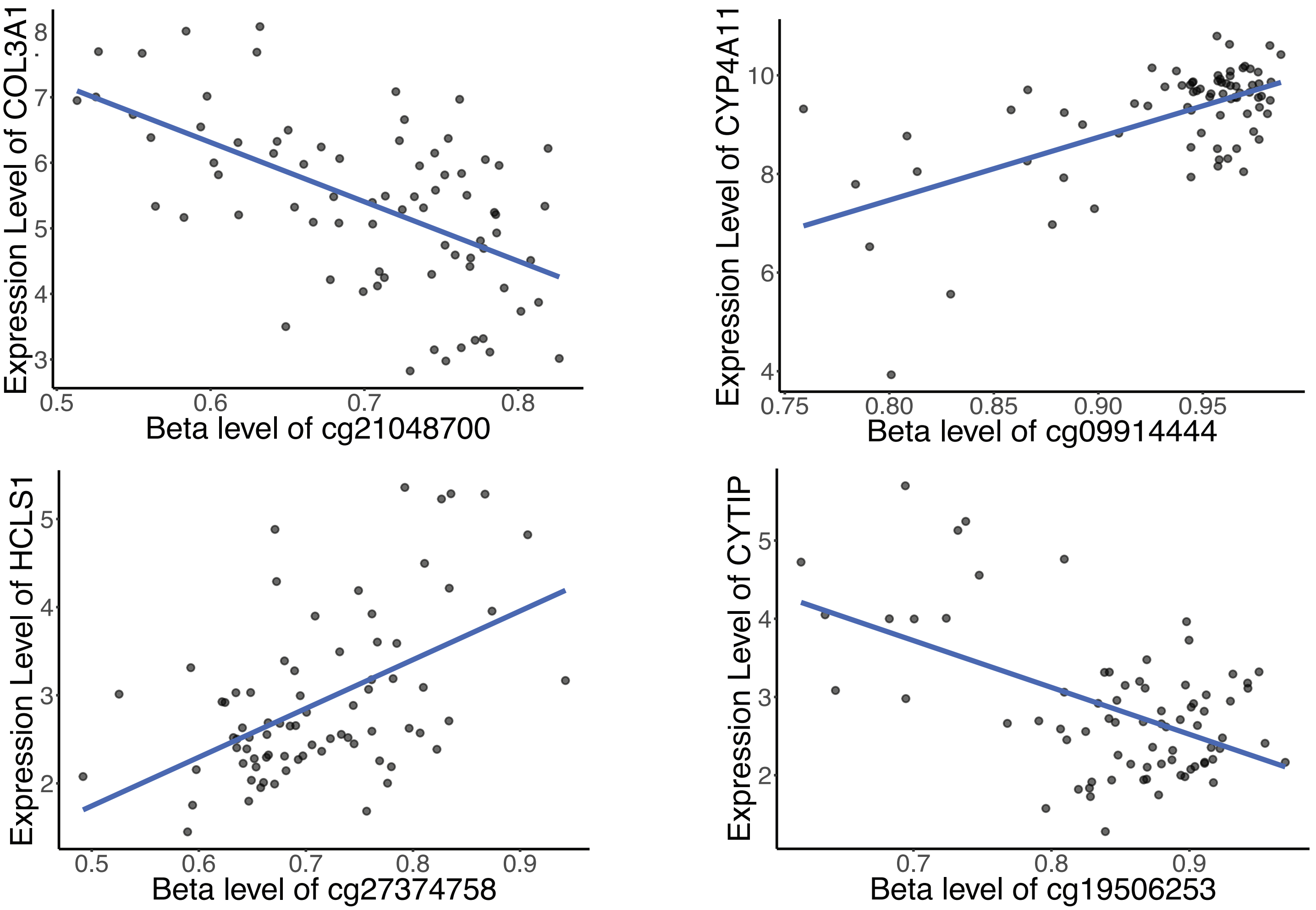

Supplementary Figure 11. Correlation of top probes that improve CKD progression model methylation levels with Cis-gene expression levels.

Supplementary Figure 12.

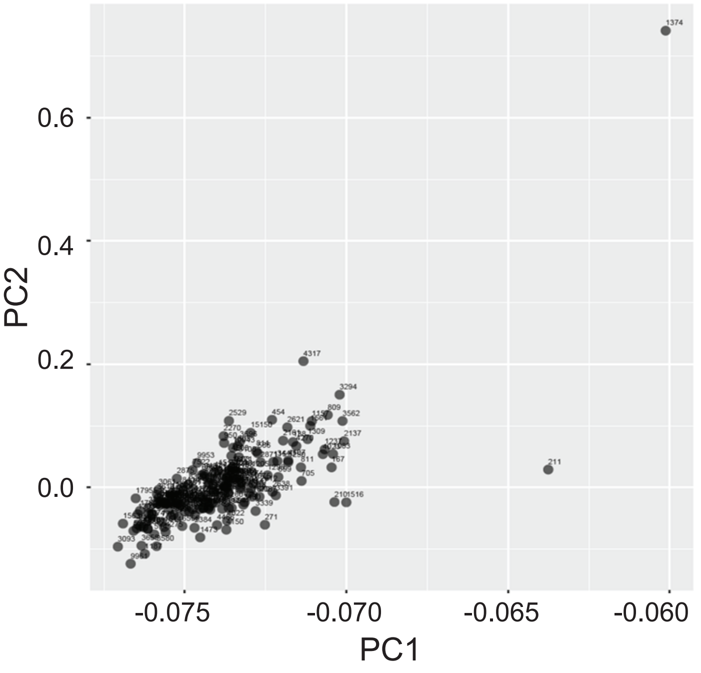

Supplementary Figure 12. Principal Component Analysis for the replication blood cohort (n=115).

Supplementary Table 1. Clinical variables associated with degree of interstitial fibrosis

Variable	Correlation Coefficient*	P-value*
Baseline eGFR	-0.6370148	7.31E-11
Diabetes	0.463479	9.00E-06
Hypertension	0.2787701	0.01071
Mean Arterial Pressure (MAP)	0.07641616	0.5295
Systolic BP	0.1475063	0.2196
Urine Dipstick (Albuminuria)	0.5419931	1.74E-07
Age	0.006002272	0.9568
Weight	-0.03595815	0.75
Height	-0.2027584	0.06947
Body Mass Index	0.04058141	0.7225
Sex	-0.03815405	0.7304
Race	0.01945298	0.8606

* Univariate analysis utilizing two sided Pearson correlation

Supplementary Table 2. Demographic and clinical characteristics of replication data cohorts

	Replication Cohort (kidney)	Replication Cohort (blood)
Subjects (n)	N=85	N= 115
Baseline eGFR (ml/min per 1.73m^2)	56.8 (36.4)	85.4 (27.2)
Female	38 (45%)	74 (64%)
Age	59.4 (18.0)	52.3 (11.6)
Race		
Asian	2 (2%)	
Caucasian	6 (7%)	
African American	62 (72%)	
Hispanic	2 (2%)	
Multiracial	9 (11%)	
Unknown	4 (5%)	
American Indian	NA	115 (100%)
Diabetes	21 (25%)	115 (100%)
duration (years)	NA	17.5 (6.6)
Hemoglobin A1C (for DM)	7.0 (1.7)	10.1 (2.1)
Hypertension	68 (80%)	NA
mean blood pressure (mmHg)	101.6 (14.6)	99.3 (10.9)
Proteinuria: dipstick (0-5)	1.5 (1.6)	NA
Albumin-creatinine ratio (mg/g)	NA	1802.7 (2298.0)
BMI (kg/m^2)	28.4 (7.8)	NA
End Stage Renal Disease	NA	N=45
Subjects with longitudinal eGFR data (n)	N= 0	N= 115
Time span (years)		5.6 (3.5)
Unadjusted GFR Slope (ml/min per 1.73m^2 per year)		-6.5 (6.5)
Adjusted GFR Slope (ml/min per 1.73m^2 per year)		-5.8 (3.3)

Data are mean (SD) or n (%)

Supplementary Table 3. Histological characteristics of replication cohort

	Replication Cohort
n	75
Hypoperfused Glomeruli (0-3)	0.77 (0.88)
Glomerular Wall Thickening (0-3)	0.30 (0.68)
Mesangial Matrix (0-3)	0.45 (0.77)
Mesangial Cellularity (0-3)	0.25 (0.60)
KW Nodule (0-1)	0.01 (0.11)
Pericapsular Fibrosis (0-2)	0.94 (0.84)
Globally Sclerotic Glomeruli (%)	25.31 (30.88)
Segmentally Sclerotic Glomeruli (%)	1.16 (3.46)
Tubular Atrophy (%)	28.89 (35.50)
Acute Tubular Injury (%)	3.57 (12.52)
Tubules Reabsorption (0-3)	0.18 (0.46)
Interstitial Fibrosis (%)	27.62 (32.40)
Plasmacytic Infiltrate (0-3)	0.54 (0.69)
Lymphocytic Infiltrate (0-3)	1.24 (0.82)
Eosinophilic Infiltrate (0-3)	0.22 (0.48)
Vessel Medial Thickening (0-3)	0.22 (0.60)
Vessel Intimal Fibrosis (0-3)	1.78 (1.01)
Vessel Arteriolar Hyalinosis (0-3)	0.52 (0.74)

Data are mean (SD)

Supplementary Table 4. Gene Ontology for top methylation probes associated with degree of interstitial fibrosis

Category	Term	RT	Genes	Count	%	P-Value	Benjamini
GOTERM_BP_1	Biological adhesion	RT		11	22.4	5.60E-03	1.10E-01
GOTERM_BP_1	localization	RT		23	46.9	7.40E-03	7.10E-02

!

Supplementary Table 5. Demographic and clinical characteristics of sub-cohorts

	Primary and Replication Cohort (kidney) with Gene Expression Data	Primary Cohort (kidney) with longitudinal eGFR Data
Subjects (n)	N=77	N=69
Baseline eGFR (ml/min per 1.73m^2)	67.5 (26.4)	66.3 (24.7)
Female	34 (44%)	38 (55%)
Age	64.9 (11.1)	64.7 (11.5)
Race		
Asian	3 (4%)	1 (1%)
Caucasian	17 (22%)	13 (19%)
African American	24 (31%)	26 (38%)
Hispanic	5 (6%)	6 (9%)
Multiracial	14 (18%)	13 (19%)
Unknown	14 (18%)	10 (15%)
Diabetes	37 (48%)	31 (45%)
Hypertension	55 (71%)	52 (75%)
MAP	94.5 (11.6)	93.4 (11.7)
Proteinuria: dipstick (0-5)	1 (1.5)	0.9 (1.4)
BMI (kg/m^2)	31.0 (9.6)	30.7 (10.0)

Data are mean (SD) or n (%)

Supplementary Table 6. Longitudinal follow up data by chronic kidney disease (CKD) stage

CKD stage	GFR (ml/min per 1.73m^2)	Age (years)	% Interstitial Fibrosis	Unadjusted GFR slope (ml/min per 1.73m2 per year)	Adjusted GFR slope (ml/min per 1.73m2 per year)	n
0	115.1 (13.1)	55 .3 (6.2)	4.67 (4.62)	1.0 (3.4)	-1.4 (0.8)	4
1	98.2 (2.5)	56.0 (8.9)	3.3 (2.9)	-1.3 (4.7)	-2.5 (1.3)	3
2	73.3 (8.4)	65.1 (11.7)	6.3 (6.6)	-6.4 (5.7)	-4.1 (1.0)	43
3	44.4 (8.2)	71.0 (9.0)	22.0 (22.9)	-7.9 (4.1)	-5.3 (1.0)	13
4	19.4 (2.7)	64.0 (14.9)	45.0 (18.0)	-11.3 (8.0)	-6.0 (0.1)	3
5	9.7 (5.3)	54.0 (8.0)	56.6 (44.8)	-0.1 (0.4)	-4.6 (0.3)	3

Data are mean (SD)

Supplementary Table 7. Variables associated with eGFR slope

Variable	Correlation Coefficient*	P-value*
Baseline eGFR	0.6428609	2.59E-09
Diabetes	-0.5713708	2.94E-07
Hypertension	-0.2871806	0.01673
Mean Arterial Pressure (MAP)	0.129615	0.3194
Systolic BP	-0.07372888	0.5723
Urine Dipstick (Albuminuria)	-0.3493811	0.003497
Age	-0.3703063	0.001737
Weight	-0.004508233	0.9711
Height	0.2931494	0.01606
Body Mass Index	-0.1022956	0.4212
Sex	-0.02069567	0.866
Race	-0.09740657	0.4259
Hypoperfused Glomeruli (0-3)	-0.2170649	0.08
Glomerular Wall Thickening (0-3)	-0.1270326	0.3094
Mesangial Matrix (0-3)	-0.3223249	0.008831
Mesangial Cellularity (0-3)	-0.2293743	0.06394
KW Nodule (0-1)	-0.1182092	0.3445
Pericapsular Fibrosis (0-2)	-0.3310049	0.006634
Globally Sclerotic Glomeruli (%)	-0.1320976	0.3144
Segmentally Sclerotic Glomeruli (%)	-0.01834522	0.8838
Tubular Atrophy (%)	-0.280921	0.02232
Acute Tubular Injury (%)	-0.2301258	0.06516
Tubules Reabsorption (0-3)	-0.2140474	0.08944
Interstitial Fibrosis (%)	-0.2928217	0.01703
Plasmacytic Infiltrate (0-3)	-0.1514714	0.2284
Lymphocytic Infiltrate (0-3)	-0.3039231	0.01384
Eosinophilic Infiltrate (0-3)	-0.2120453	0.08994
Vessel Medial Thickening (0-3)	-0.07113111	0.5765
Vessel Intimal Fibrosis (0-3)	-0.01431152	0.9106
Vessel Arteriolar Hyalinosis (0-3)	-0.1099817	0.3794

* Univariate analysis utilizing two sided Pearson correlation

Supplementary Table 8. Progression model with replicated fibrosis-associated probes

Variable[a]	Model[b]			% Explained by Variable[c]		
	Base	Base + Probe cg00355019 (A)	Base + Probe cg07830160 (B)	Base Model	Model A	Model B
Baseline GFR	0.03***	0.04***	0.04***	20.67	31.41	30.17
Diabetes	- 0.72*	- 1.11***	- 1.05***	5.75	9.51	8.40
Age	- 0.03	- 0.01	- 0.01	6.16	0.85	0.69
CpG probe	NA	- 1.17***	-1.55***	NA	13.19	11.89
Methylation Batch	NA	NA	NA	NA	8.49	13.02
Bisulfite conversion	NA	0.42	1.96	NA	0.00	0.03
R2	0.51	0.70	0.70			
Adjusted R2	0.49	0.64	0.63			
Akaike Information Criterion	206.1	189.6	191.1			
P-value	3.13e-10	6.33e-11	1.10e-10			

a. For each variable, coefficient estimates are shown with the following significance codes: 0 ‘***’; 0.001 ‘**’; 0.01 ‘*’; 0.05 ‘·’ .
b. Model is a weighted linear regression model of adjusted eGFR slope (weight = inverse variance of adjusted eGFR slope). Base model includes variables: baseline eGFR, Diabetes, and Age. Models A and B include base variables with the addition of methylation level at probe location, methylation batch, and bisulfite conversion efficiency.
c. Proportion of variance explained by the variable based on conditional sum of squares calculated in Type II ANOVA analysis.

Supplementary Table 9. Gene Ontology for top methylation probes that improve model of kidney function

Category	Term	RT	Genes	Count	%	P-Value	Benjamini
GOTERM_BP_1	developmental process	RT		131	40.6	3.70E-05	8.50E-04
GOTERM_BP_1	biological adhesion	RT		47	14.6	1.50E-03	1.70E-02
GOTERM_BP_1	signaling	RT		134	41.5	1.60E-03	1.20E-02
GOTERM_BP_1	response to stimulus	RT		169	52.3	2.50E-03	1.40E-02
GOTERM_BP_1	single-organism process	RT		250	77.4	3.80E-03	1.80E-02
GOTERM_BP_1	localization	RT		124	38.4	4.80E-03	1.80E-02
GOTERM_BP_1	regulation of biological process	RT		209	64.7	8.70E-03	2.80E-02
GOTERM_BP_1	biological regulation	RT		219	67.8	9.50E-03	2.70E-02
GOTERM_BP_1	multicellular organismal process	RT		142	44	1.10E-02	2.80E-02
GOTERM_BP_1	cellular component organization or biogenesis	RT		125	38.7	3.10E-02	7.00E-02
GOTERM_BP_1	growth	RT		25	7.7	3.40E-02	6.90E-02
GOTERM_BP_1	immune system process	RT		54	16.7	5.10E-02	9.60E-02
GOTERM_BP_1	locomotion	RT		35	10.8	7.60E-02	1.30E-01
GOTERM_BP_1	cellular process	RT		276	85.4	8.90E-02	1.40E-01

!
