# Supplementary material for: Kidney cytosine methylation changes improve renal function decline estimation in patients with diabetic kidney disease
Source: Nat Commun. 2019 Jun 5;10:2461. doi: 10.1038/s41467-019-10378-8 (PMC6549146; doi:10.1038/s41467-019-10378-8)
Supplement: Supplementary file 3 — Description of Additional Supplementary Files [file 41467_2019_10378_MOESM3_ESM.docx]

**Description of Additional Supplementary Files**

**File Name: Supplementary Data 1**

**Description:** *Top 65 probes significantly associated with interstitial fibrosis and passed independent replication*. (a) Association determined by linear regression models adjusted for age, gender, race, diabetes, hypertension, batch, bisulfite conversion, and degree of lymphocytic infiltrate on histology.

**File Name: Supplementary Data 2**

**Description:** *Methylation probes for which methylation level correlates with nearby (cis) gene expression level.* (a) Association determined by linear regression. Significance determined by random permutation method (cutoff p-value = 8e-5).

**File Name: Supplementary Data 3**

**Description:** *Top 471 probes that improve model of kidney function declineusing weighted regression.* (a) Model is a weighted linear regression model of adjusted eGFR slope (weight = inverse variance of adjusted eGFR slope). Base model includes variables: baseline eGFR, Diabetes, and Age (base model AIC = 206). When methylation level of probe is added to base model, the following variables are also added: methylation batch, and bisulfite conversion efficiency. (b) Association determined by linear regression models adjusted for age, gender, race, diabetes, hypertension, batch, bisulfite conversion, and degree of lymphocytic infiltrate on histology.

**File Name: Supplementary Data 4**

**Description:** *Top 67 probes that improve model of kidney function decline using weighted regression when hypertension is added to model*. (a) Model is a weighted linear regression model of adjusted eGFR slope (weight = inverse variance of adjusted eGFR slope). Base model includes variables: baseline eGFR, Diabetes, and Age (base model AIC = 206). When methylation level of probe is added to base model, the following variables are also added: methylation batch, and bisulfite conversion efficiency. (b) Association determined by linear regression models adjusted for age, gender, race, diabetes, hypertension, batch, bisulfite conversion, and degree of lymphocytic infiltrate on histology.

**File Name: Supplementary Data 5**

**Description:** *Top probes that improve progression model and are associated with gene expression changes*. (a) Model is a weighted linear regression model of adjusted eGFR slope (weight = inverse variance of adjusted eGFR slope). Base model includes variables: baseline eGFR, Diabetes, and Age (base model AIC = 206). When methylation level of probe is added to base model, the following variables are also added: methylation batch, and bisulfite conversion efficiency. (b) Association determined by linear regression models adjusted for age, gender, race, diabetes, hypertension, batch, bisulfite conversion, and degree of lymphocytic infiltrate on histology. (c) Association determined by linear regression. Significance determined by random permutation method (cutoff p-value = 8e-5).

**File Name: Supplementary Data 6**

**Description:** *Probe look-up for 341 methylation probes identified by Chen et al.* (a) Model is a weighted linear regression model of adjusted eGFR slope (weight = inverse variance of adjusted eGFR slope). Base model includes variables: baseline eGFR, Diabetes, and Age (base model AIC = 206). When methylation level of probe is added to base model, the following variables are also added: methylation batch, and bisulfite conversion efficiency. (b) Association determined by linear regression models adjusted for age, gender, race, diabetes, hypertension, batch, bisulfite conversion, and degree of lymphocytic infiltrate on histology.
