# Supplementary material for: Kidney cytosine methylation changes improve renal function decline estimation in patients with diabetic kidney disease
Source: Nat Commun. 2019 Jun 5;10:2461. doi: 10.1038/s41467-019-10378-8 (PMC6549146; doi:10.1038/s41467-019-10378-8)
Supplement: Supplementary file 10 — Reporting Summary [file 41467_2019_10378_MOESM10_ESM.pdf]

nature research

Corresponding author(s): Katalin Susztak

Last updated by author(s): 4/15/2019

Reporting Summary

Nature Research wishes to improve the reproducibility of the work that we publish. This form provides structure for consistency and transparency in reporting. For further information on Nature Research policies, see Authors & Referees and the Editorial Policy Checklist.

Statistics

For all statistical analyses, confirm that the following items are present in the figure legend, table legend, main text, or Methods section.

n/a	Confirmed	
☐	☒	The exact sample size (*n*) for each experimental group/condition, given as a discrete number and unit of measurement
☒	☐	A statement on whether measurements were taken from distinct samples or whether the same sample was measured repeatedly
☐	☒	The statistical test(s) used AND whether they are one- or two-sided<br>*Only common tests should be described solely by name; describe more complex techniques in the Methods section.*
☐	☒	A description of all covariates tested
☐	☒	A description of any assumptions or corrections, such as tests of normality and adjustment for multiple comparisons
☐	☒	A full description of the statistical parameters including central tendency (e.g. means) or other basic estimates (e.g. regression coefficient) AND variation (e.g. standard deviation) or associated estimates of uncertainty (e.g. confidence intervals)
☐	☒	For null hypothesis testing, the test statistic (e.g. *F*, *t*, *r*) with confidence intervals, effect sizes, degrees of freedom and *P* value noted<br>*Give P values as exact values whenever suitable.*
☒	☐	For Bayesian analysis, information on the choice of priors and Markov chain Monte Carlo settings
☒	☐	For hierarchical and complex designs, identification of the appropriate level for tests and full reporting of outcomes
☐	☒	Estimates of effect sizes (e.g. Cohen's *d*, Pearson's *r*), indicating how they were calculated

Our web collection on statistics for biologists contains articles on many of the points above.

Software and code

Policy information about availability of computer code

Data collection	*Provide a description of all commercial, open source and custom code used to collect the data in this study, specifying the version used OR state that no software was used.*
Data analysis	Data was analyzed using the following R packages: minfi (version 1.28.0), lme4 (version 1.1-12), glmnet (version 2.0-5), Rnbeads (version 2.0.0), as well as ChromHMM (version 1.12)

For manuscripts utilizing custom algorithms or software that are central to the research but not yet described in published literature, software must be made available to editors/reviewers. We strongly encourage code deposition in a community repository (e.g. GitHub). See the Nature Research guidelines for submitting code & software for further information.

Data

Policy information about availability of data

All manuscripts must include a data availability statement. This statement should provide the following information, where applicable:

- Accession codes, unique identifiers, or web links for publicly available datasets
- A list of figures that have associated raw data
- A description of any restrictions on data availability

The methylation data is available at GEO: GSE50874 [https://www.ncbi.nlm.nih.gov/geo/query/acc.cgi?acc=GSE50874].The gene expression data is available at ArrayExpress: E-MTAB-5929, E-MTAB-2502 [https://www.ebi.ac.uk/arrayexpress/experiments/E-MTAB-5929/, https://www.ebi.ac.uk/arrayexpress/experiments/E-MTAB-2502/]. The rest of the data are available from the corresponding author upon reasonable request.

Field-specific reporting

Please select the one below that is the best fit for your research. If you are not sure, read the appropriate sections before making your selection.

☒ Life sciences ☐ Behavioural & social sciences ☐ Ecological, evolutionary & environmental sciences

For a reference copy of the document with all sections, see nature.com/documents/nr-reporting-summary-flat.pdf

Life sciences study design

All studies must disclose on these points even when the disclosure is negative.

Sample size	Primary cohort n=91.
Data exclusions	For the primary cohort analysis we removed one outlier sample (based on principal component analysis). For the longitudinal analysis, we excluded subjects without a minimum 3 eGFR estimations and one eGFR estimation at least 3 months post-nephrectomy. Subjects with unadjusted eGFR slope < -40 or >40 ml/min per 1.73m2 per year were excluded. For the cytosine methylation analysis, we excluded probes in close proximity to regions with genetic variations (using the Gap Hunter package), probes located on sex chromosomes and those known to cross-hybridize to other locations as well as those with poor detection p-value (p>0.01).
Replication	We replicated our findings in two independent cohorts. The first replication cohort included kidney methylomes (n=85). The second replication cohort used blood methylomes (n=115).
Randomization	For linear regression analysis (association of methylation with interstitial fibrosis), we controlled for age, sex, race, diabetes, hypertension, batch, bisulfite conversion, and degree of lymphocytic infiltrate on histology. For the CKD progression model, we controlled for age, baseline eGFR, diabetes, batch, and bisulfite conversion -- this model was weighted by the inverse variance of eGFR slope.
Blinding	Blinding was not relevant or this type of analysis.

Reporting for specific materials, systems and methods

We require information from authors about some types of materials, experimental systems and methods used in many studies. Here, indicate whether each material, system or method listed is relevant to your study. If you are not sure if a list item applies to your research, read the appropriate section before selecting a response.

Materials & experimental systems

n/a	Involved in the study
☒	☐ Antibodies
☒	☐ Eukaryotic cell lines
☒	☐ Palaeontology
☒	☐ Animals and other organisms
☐	☒ Human research participants
☒	☐ Clinical data

Methods

n/a	Involved in the study
☒	☐ ChIP-seq
☒	☐ Flow cytometry
☒	☐ MRI-based neuroimaging

Human research participants

Policy information about studies involving human research participants

Population characteristics	Kidney samples were collected at Albert Einstein College of Medicine Montefiore Medical Center between the years of 2007-2011. Samples were collected during nephrectomy procedures. Samples were de-identified and corresponding clinical information was collected by an honest broker.
Recruitment	Subjects were not recruited.
Ethics oversight	Institutional Review Boards at the Albert Einstein College of Medicine Montefiore Medical Center (IRB 2002-202) and the University of Pennsylvania (IRB 815796) reviewed this study. This project utilized de-identified kidney biospecimens. Therefore, this project does not meet the definition of human subject research and IRB review was not required. It was completed in compliance with all relevant ethical regulations.

Note that full information on the approval of the study protocol must also be provided in the manuscript.
